# Spleen metastases secondary to gastric neuroendocrine carcinoma: case report

**DOI:** 10.11604/pamj.2022.42.131.34091

**Published:** 2022-06-16

**Authors:** Sahar Nasr, Amal Khsiba, Moufida Mahmoudi, Asma Ben Mohamed, Mehdi Bouasida, Lamine Hamzaoui

**Affiliations:** 1 Faculty of Medicine of Tunis, Tunis, Tunisia

**Keywords:** Spleen, metastases, neuroendocrine carcinoma, stomach, case report

## Abstract

Neuroendocrine carcinoma (NEC) in the stomach represents a rare and rapidly growing type of gastric tumors. They are considered a distinct entity of neuroendocrine tumors characterized by an aggressive behavior and high metastases rate. On the other hand, spleen metastases of neuroendocrine tumors are extremely rare. We report the first case of spleen metastases of gastric neuroendocrine carcinoma. The patient was a 54-year-old male who presented with a 10-month history of epigastralgia. Upper gastro-intestinal endoscopy revealed a 5 cm ulcerative lesion located in the greater gastric curvature. Biopsies with immunohistochemical staining revealed gastric neuroendocrine carcinoma. Abdominal computed tomography showed thickening of the stomach with two large solid spleen lesions. Abdominal Magnetic Resonance Imaging and 18-fluorodexyglucose positron-emission tomography revealed peritoneal carcinosis and splenic metastases with splenic vein invasion. Clinicians should keep in mind that splenic metastases can arise from gastric neuroendocrine tumors (NETs).

## Introduction

Neuroendocrine carcinoma (NEC) in the stomach represents a rare and rapidly growing type of gastric tumors defined by a Ki-67 proliferation index of 20% or higher according to the WHO 2010 definition [1]. NEC are considered a distinct entity of neuroendocrine tumors (NETs) owing to their aggressive behavior and high metastases rate. Inup to 85% of cases, NEC are diagnosed at an advanced stage with an overall survival ranging from 5 to 38 months [2]. The most commonly involved metastases site is the liver (70%) followed by the lung and bones (15%). We report a very rare case of splenic metastases originating from a gastric neuro-endocrine carcinoma.

## Patient and observation

**Patient information:** a 54-year-old patient presented with a prandial epigastralgia persisting for over 10 months. The patient was an active smoker with an unremarkable medical history. He denied weighed loss, asthenia and melena.

**Clinical findings:** physical examination revealed tenderness in the epigastric region and the left upper quadrant.

**Figure 1:**
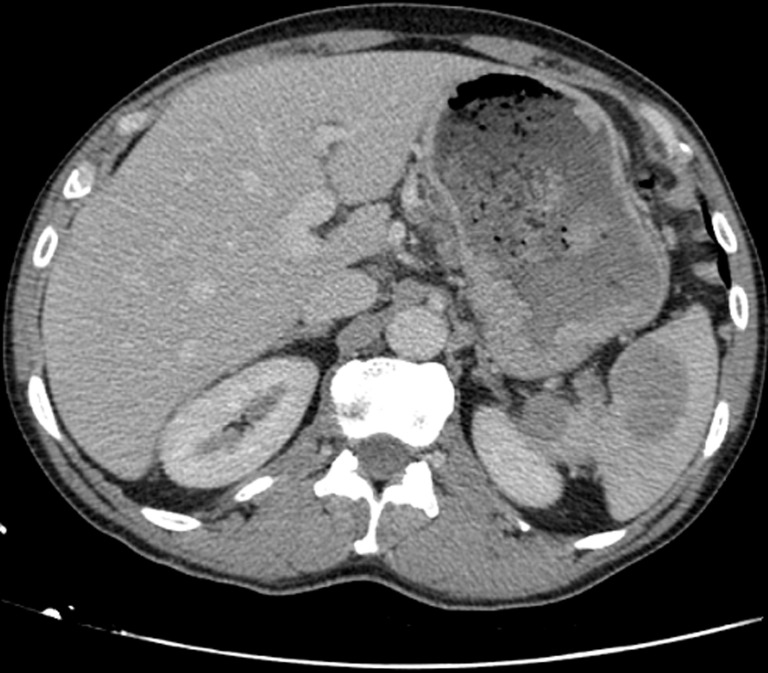
abdominal CT scan findings

**Figure 2:**
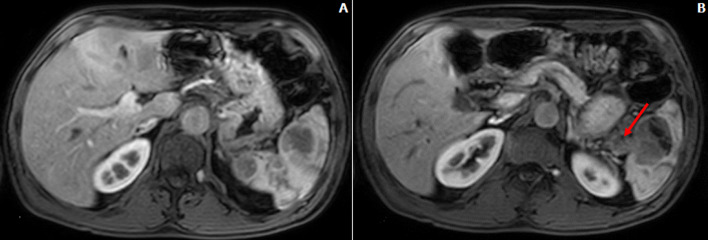
(A,B) abdominal MRI findings

**Diagnostic assessment:** upper gastrointestinal (GI) endoscopy revealed a 5 cm ulcerative lesion located in the greater gastric curvature with a centre situated 4 cm below the gastroesophageal junction. A pathological review of the gastric lesion showed nests of poorly differentiated tumoral cells. Immunohistochemical staining was focally and moderately positive for synaptophysin and CD56 and negative for chromogranine A. The Ki-67 labelling index was 90%. Based on these findings, the patient was diagnosed with gastric neuroendocrine carcinoma (NEC). The computed tomography (CT) scan of the abdomen and the pelvis revealed thickening of the stomach wall with no signs of extension to the surrounding soft tissue and enlarged lymph nodes in the left perigastric and the pericardial areas. The spleen had a normal size and contained two contrast-enhancing large lesions with cystic components measuring 30x30 mm and 45x40 mm (Figure 1). An abdominal Magnetic Resonance Imaging (MRI) was performed to further evaluate the splenic lesions and to look for hepatic metastases. The splenic lesions were isointense on T1-weighted MRI, and mildly hyperintense on T2-weighted MRI with heterogeneous enhancement and protrusion into the splenic vein (Figure 2). The MRI also revealed three omental masses (7-8 mm) that appeared hyperintense on diffusion-weighted MRI. On an 18-fluorodeoxyglucose positron-emission tomography (FDG-PET) scan, intense FDG uptake was found in the greater gastric curvature, the spleen, the perigastric lymph nodes and the peritoneum (right iliac fossa and the right colon angle).

**Diagnosis:** these results confirmed the diagnosis of NEC with peritoneal carcinosis and splenic metastases.

**Therapeutic interventions**

**Follow-up and outcome of interventions:** the patient received combination chemotherapy with cisplatin and etoposide.

**Patient perspective:** the patient is currently receiving chemotherapy. Thus, it is too soon for us to evaluate the response to treatment.

**Informed consent:** written consent was obtained from the patient to publish this case.

## Discussion

The incidence of neuroendocrine tumors in the gastro-entero-pancreatic tract has been steadily increasing during the last decades owing to the improvements in imaging modalities [3]. Yet, the rate of metastases at the time of diagnosis is still high ranging from 50% to 85% [3].

Our patient had peritoneal carcinosis and metastases in the spleen without any other visceral involvement. In fact, synchronous and metachronous spleen metastases are uncommon in solid tumors [4]. This is mainly explained by the constant splenic blood flow, the rhythmic capsule movements and the absence of afferent lymphatic capillaries. In addition, the splenic macrophages of the reticulo-endothelial system create a hostile environment for tumor cells implantation and proliferation [5]. Furthermore, it has been speculated that the sharp angle of the splenic artery would make it difficult for tumoral emboli to reach the spleen [6]. The most commonly involved primary tumors of splenic metastases are lung, breast carcinoma and skin melanoma. Regarding gastric tumors, a Chinese study found that gastric adenocarcinoma was the primary source of spleen nonlymphoid metastases [4]. On the other hand, only three cases of spleen metastases associated with gastroenteropancreatic NETs were to our knowledge reported despite an extensive review of the literature (two cases of pancreatic NETs and one grade 1 gastric NETs) [7]. In a large nationwide study, gastric NETs were reported to be less likely to metastasize when compared to adenocarcinoma with the liver being the main secondary location as it was involved in 84% of cases [8]. As for spleen metastases, they remain subject to speculation. Some authors speculate that they arise from direct invasion of gastric carcinomas as a part of peritoneal seeing owing to the organ's anatomical contiguity. Yet, this theory does not apply to this case as there was more than one splenic lesion with no signs of extension of the gastric NEC to the surrounding tissue and no generalized peritoneal carcinomatosis on both CT scan and MRI. Interestingly, the splenic nodules in our case were massive while the peritoneal involvement was limited to three infracentimetric nodules on abdominal MRI. Another popular theory is the hematogenous origin through either the splenic artery or vein. Marymont and colleagues have found in a histological study of metastatic spleens several cases with involvement of venous sinusoids and/or the red pulp and extensive nests of intravascular tumoral cells, which is highly suggestive of a hematogenous origin. Yet, this mechanism implies systemic circulation of tumor cells which would give rise to metastases in other organs. On the other hand, the splenic vein obstruction might explain the splenic focus of metastases. In fact, it has been suggested that voluminous gastric tumors might affect portal vein flow resulting in splenic vein congestion and possibly tumor cell implantation in the spleen [9].

Splenic metastases can be challenging to diagnose. In our case, we were not able to characterize the lesions based on the CT scan findings. The radiological appearance of splenic metastases is very variable. The lesions might be solitary, numerous or diffuse with different sizes and splenic locations [10]. On CT scan, the lesions are usually well-defined with peripheral enhancement and no calcifications are usually detected while they appear as T2 hyperintense and T1 hypo to isointense lesions on MRI [11,12].

## Conclusion

This is the first report of splenic metastases of gastric neuroendocrine carcinoma and the second one in gastric NETs combined. Clinicians should keep in mind that splenic metastases can arise from gastric NETs. Further evaluation with MRI or PET/CT with 18-fluorodeoxyglucose is required when solid splenic lesions are diagnosed in patients with gastric NETs.
